# Exposure to Nitrogen Oxide in the First Trimester and Risk of Cardiovascular-Related Malformations: A Dose-Response Meta-Analysis of Observational Studies

**DOI:** 10.1155/2018/1948407

**Published:** 2018-04-10

**Authors:** Tie-Ning Zhang, Da Li, Qi-Jun Wu, Jing Xia, Ri Wen, Xing-Chen Chen, Ni Yang, Yan-Ling Chen, Yan-Hong Huang, Chun-Feng Liu

**Affiliations:** ^1^Department of Pediatrics, Shengjing Hospital of China Medical University, Shenyang, China; ^2^Department of Obstetrics and Gynecology, Shengjing Hospital of China Medical University, Shenyang, China; ^3^Department of Clinical Epidemiology, Shengjing Hospital of China Medical University, Shenyang, China; ^4^Department of Neurosurgery, Shengjing Hospital of China Medical University, Shenyang, China; ^5^Liaoning Women and Children's Health Hospital, Shenyang, China; ^6^Department of Science and Education, Shenyang Women and Children Health Care Centre, Shenyang, China

## Abstract

Nitrogen oxide (NO_*x*_) is produced during combustion at high temperature, which is a major constituent of air pollutants. Recent studies suggested inconsistent results on the association between NO_*x*_ exposure and cardiovascular-related malformations. We aimed to assess aforementioned association in pregnant women in the first trimester and cardiovascular-related malformations of infants. A systematic literature review identified studies for observational studies about NO_*x*_ exposure and cardiovascular-related malformation in PubMed. Random-effect models were used to estimate summary odds ratio (SOR) and 95% confidence intervals (CIs) for aforementioned association. Finally, nine studies met the inclusion criteria. Overall, the SOR of cardiovascular-related malformation per 10 ppb increment in NO_*x*_ and NO_2_ concentration was 1.01 (95% CI: 0.98–1.04; *I*^2^ = 38.6%, *P* = 0.09) and 0.99 (95% CI: 0.95–1.04; *I*^2^ = 37.8%, *P* = 0.13), respectively. Stratifying by study design, geographic locations, and confounded adjustments, the majority of strata showed negative results, which were consistent with the main findings. However, we found that exposure to NO_*x*_ and NO_2_ in the first trimester increased the risk of coarctation of the aorta (COA) malformation by 13% and 19%, respectively. Our study provided limited evidence regarding the association between NO_*x*_ exposure in the first trimester and cardiovascular-related malformations in infants.

## 1. Introduction

Air pollution has become a major problem in many countries. Increasing evidence showed that air pollution could not only lead to global warming, but also have adverse effects on the fetus and newborn from maternal exposure [1, 2], such as preterm birth, low birth weight, and intrauterine growth retardation [3–6]. Of note, nitrogen oxide (NO_*x*_), a major constituent of air pollutants, mainly refers to nitric oxide (NO) and nitrogen dioxide (NO_2_), which are produced during combustion, especially at high temperature. These two substances are important trace species in earth's atmosphere.

Congenital malformations are an important cause of infant mortality and a leading cause of disability worldwide [7]. Notably, cardiovascular-related malformation, which constitutes most common group of birth defects [8], has become the main cause of death in infants with congenital anomalies and is associated with a considerable burden on public [9–12]. For example, the previous study reported that congenital heart defects occur in 4–8 of 1000 live births [13], the most common being ventricular septal defects (VSD) (27.5/10,000 live births) and atrial septal defects (ASD) (10.6/10,000 live births) [14]. Although the etiology of cardiovascular-related malformation is still unclear, several studies suggested that it might be related to both genetic and environmental factors [10, 15]. Additionally, recent studies have proposed that air pollution including nitrogen oxide could play an important role in causing cardiac defect [2, 16], because of its ability to act directly as prooxidants of lipid and proteins or as free radical generators promoting oxidative stress and the induction of inflammatory responses [17]. For example, a meta-analysis suggested that NO_2_ exposure was related to increases in risk of coarctation of the aorta (COA) and tetralogy of Fallot (TOF) [18], while the other one suggested that NO_2_ exposure was only associated with COA [19]. However, several limitations were observed in the previous meta-analyses: (1) the exposure period of NO_*x*_ of pregnant women was not consistent; for example, the meta-analysis carried out by Vrijheid et al. [18] included different exposure periods of NO_*x*_ (e.g., first trimester, second trimester, third trimester, and three-month preconception); (2) the results of subgroup analyses stratified by adjustment for potential confounders were limited; (3) although atrial septal defect (ASD) and ventricular septal defect (VSD) are the most common cardiovascular-related malformations [14], the previous meta-analyses did not perform throughout analyses regarding these two malformations and the evidence of the associations between NO_*x*_ and ASD or VSD was relatively finite.

In order to better understand the relationship between nitrogen oxide exposure in pregnant women in the first trimester and cardiovascular-related malformations of infants, we performed a systematic review and meta-analysis using data from epidemiologic studies.

## 2. Methods

### 2.1. Literature Search

This meta-analysis was planned, performed, and reported in adherence to the Meta-analysis Of Observational Studies in Epidemiology (MOOSE) guideline [20]. We conducted computerized literature searches in the PubMed and reviewed the data from the database index through April 15, 2017. The following search key words and Medical Subject Heading (MeSH) terms were used: (air pollution OR nitrogen oxide OR nitrogen dioxide OR nitric oxide OR NO_*x*_ OR NO_2_ OR NO) AND (birth defect OR congenital anomalies OR cardiovascular-related malformation OR cardiac anomalies OR cardiac malformation OR congenital heart disease OR defect OR congenital abnormalities OR birth outcome OR obstetrical outcome). Additionally, the references cited in the retrieved articles were scrutinized by manual research.

### 2.2. Study Selection

The published studies were considered to be included if they met the following criteria: if they (i) used an epidemiologic study design (e.g., case-control, case-cohort, and cohort study); (ii) defined the exposure period of nitrogen oxide as the first trimester or in the range of first trimester during pregnancy; (iii) reported cardiovascular-related malformation as the outcomes of interest; (iv) reported the usable risk estimates (e.g., odd ratio, risk ratio, or relative risk with 95% confidence intervals (CIs) or necessary data for calculation) of the association between nitrogen oxide exposure and cardiovascular-related malformation.

The published studies were excluded by the following exclusion criteria: if they (i) were nonepidemiologic studies (e.g., case-control, case-cohort, and cohort study), review articles, systematic review and meta-analyses, commentaries, editorials, or meeting abstracts; (ii) reported the risk estimates that could not be summarized (such as reporting the risk estimates without 95% CIs) or used for further unit conversion (such as reporting the risk estimates of NO_*x*_ with the unit of “microgram per cubic meter (ug/m^3^)”); (iii) reported the exposure period during the period of preconception or other trimesters; (iv) were not human studies or published in English.

When duplicate articles from the same study were identified, we selected the most recent report that contained the largest number of the cases or cohort that matched our interest. All abovementioned study selection and exclusion procedures were carried out by two independent researchers (Tie-Ning Zhang and Qi-Jun Wu).

### 2.3. Data Extraction

Data was independently extracted according to a standardized format by two researchers (Tie-Ning Zhang and Jing Xia) for each eligible study. Disagreements were discussed and solved by a third researcher (Qi-Jun Wu). The following important study characteristics were abstracted from each included study: the first author, year of publication, geographic location, study period, study design, number of cases, gestational period, kind of exposure, outcome with their risk estimates, and 95% CIs. We also extracted the factors matched between cases and controls and potential confounders of each study. If there were multiple estimates of the association, we extracted the estimate that was adjusted for the largest number of potential confounders. If both single- and multiple-pollutant models were presented in a study, we selected single-pollutant model in our meta-analysis.

### 2.4. Quality Evaluation

Two independent researchers (Tie-Ning Zhang and Jing Xia) conducted the quality assessment of included studies according to Newcastle-Ottawa Scale (NOS) for cohort and case-control studies. All 8 items in the scale were applicable to our study question. The items can be divided into 3 domains (e.g., selection, comparability, and exposure/outcome). We used these NOS parameters to evaluate the studies instead of scoring them and categorizing them into high or low quality on the basis of the scores [21–23].

### 2.5. Statistical Analysis

As the absolute risk of cardiovascular-related malformation is low, we reported all results in terms of OR for simplicity. Considering there was limited evidence regarding NO and cardiovascular-related malformation, we just examined the relationship between NO_*x*_ or NO_2_ and cardiovascular-related malformation. Firstly, we converted the unit of ug/m^3^ into part per billion (ppb) in order to allow comparison of effects among different studies. Subsequently, we summarized and converted the study-specific ORs for each 10 ppb increment in NO_*x*_ or NO_2_ concentration. For conducting dose-response meta-analysis, the study-specific trend from the correlated log ORs across the categories of NO_*x*_ or NO_2_ concentrations was computed by using the generalized least-squares trend estimation method developed by Greenland and Longnecker [24] and Orsini et al. [25]. If the study results were presented as a quantitative exposure category, for conducting dose-response meta-analysis, we required information on (i) the distribution of cases and noncases and risk estimates with variance estimates for at least three quantitative exposure categories, (ii) median or mean level of these exposures in each category (if reported based on range, the mean level was calculated by averaging the lower and upper boundary; if the lowest category was open-ended, the lowest boundary was considered zero; if the highest category was open-ended, the open-ended interval length was assumed to be the same as the adjacent interval).

To examine the associations between the exposure (e.g., NO_*x*_ and NO_2_) and the outcomes of our interest, the summary odds ratio (SOR) with 95% CIs was estimated by summarizing the risk estimates of each study using the random-effect models [26]. For the study [27–31] that separately reported several kinds of outcomes but did not combine them (e.g., the risk estimates of ASD, conotruncal defects, and transposition of the great arteries), we recalculated total risk estimates as that of cardiovascular-related malformation using fixed effect model. Because several studies [7, 27–34] reported the outcomes of ASD, VSD, TOF, and COA, we extracted this data in order to calculate the SORs of these events.

Heterogeneity among studies was assessed with *I*^2^ statistics, and an *I*^2^ value greater than 50% was considered to indicate substantial heterogeneity. To investigate the possible sources of heterogeneity of main results, we performed stratified analyses by the following study features: study design (cohort study versus case-control study), geographic location (US versus others), and potential confounders considered or adjusted for in the analyses (maternal age, socioeconomic status, conception season, year of birth, infant sex, gestational age, and educational level). Heterogeneity between subgroups was evaluated by meta-regression analysis. We also performed sensitivity analyses by excluding one study at a time to explore whether results were strongly influenced by a specific study.

Finally, publication bias was evaluated through Egger et al.'s test [35] and Begg and Mazumdar's test [36]. We assumed that there was a significant statistical publication bias if *P* value is less than 0.05 for Egger's or Begg's test. All statistical analyses were performed with Stata 12.1 (StataCorp).

## 3. Results

### 3.1. Literature Search

**Figure 1:**
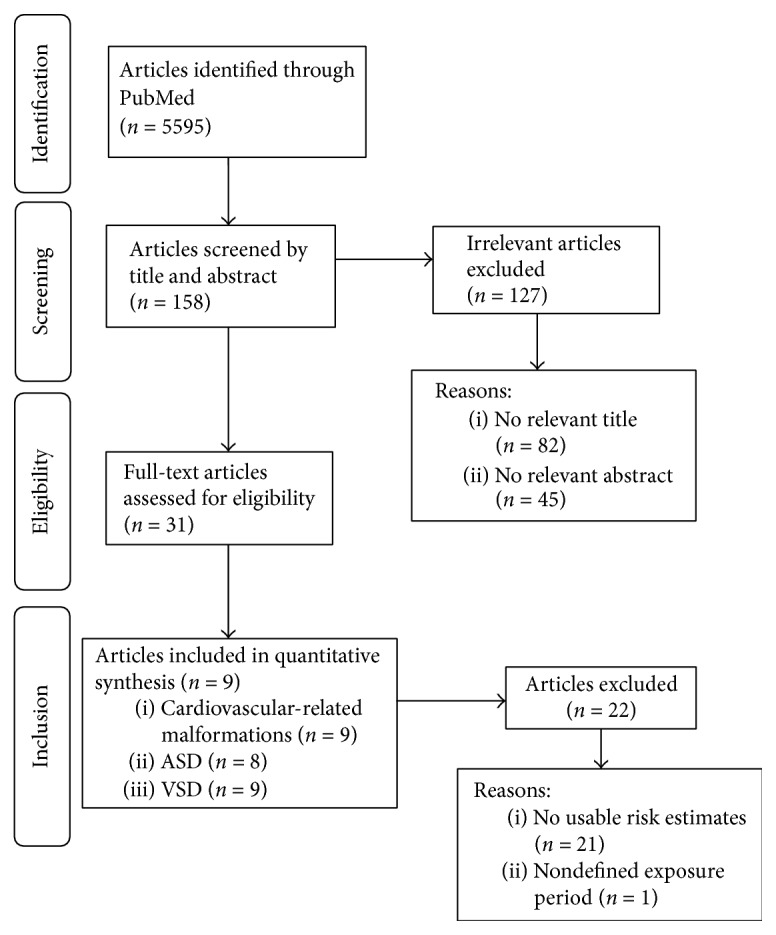
Flow chart of study selection.

Our literature search process is illustrated in Figure 1. Briefly, our initial search yielded 5,595 studies from PubMed, of which we screened the titles and abstracts. After a further review and evaluation, 5,564 studies were excluded for the general criteria. The remaining 31 studies were identified through detailed assessment. In the end, nine studies [7, 27–34] were eligible for inclusion criteria and considered into our final dose-response meta-analysis.

### 3.2. Study Characteristics

The characteristics of the eight studies are summarized in Tables S1 and S2. Briefly, these studies were published between 2009 [28, 29] and 2015 [27]. Of all these included studies, three studies each were conducted in America [29–31], two studies were conducted in Israel [7, 33], and one study was conducted in China [27], Spain [34], Northeast England [32], and Australia [28], respectively. Besides, we included three cohort studies [7, 29, 33] and six case-control studies [27, 28, 30–32, 34]. Among case-control studies, five studies [27, 28, 31, 32, 34] were population-based and another [30] was hospital-based. The number of cardiovascular-related malformations varied from 572 [28] to 4,639 [29], and the total number of cases was 19,079.

### 3.3. Quality of Included Studies

Tables S3a and S3b present the study-specific quality according to Newcastle-Ottawa quality scale. As for cohort studies, in the “follow-up long enough for outcomes to occur” and “adequacy of follow-up of cohorts” categories, no studies were assigned scores because these three cohort studies were all retrospective. The maximum score is seven [7] and the minimum score is five [29]. As for case-control studies, in the “non-response rate” categories, three studies were not assigned score because they did not refer to non-response rate or provide available data for calculation. The maximum score is nine [27] and the minimum score is six [28].

### 3.4. NO_*x*_ and Cardiovascular-Related Malformation per 10 ppb Increment

**Figure 2:**
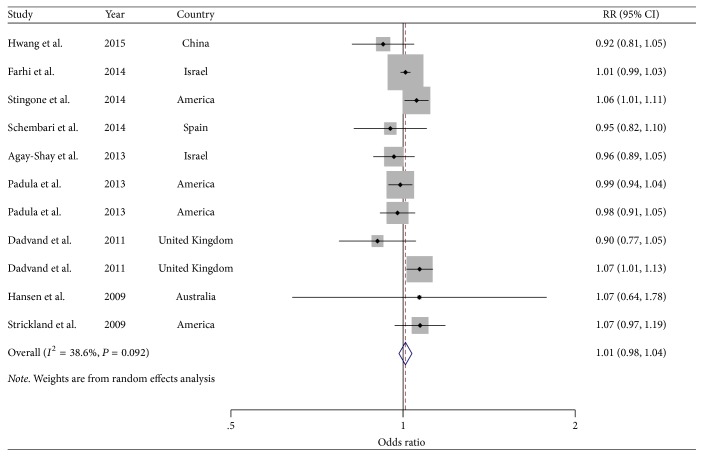
*Forest plots of the relationship between NO*_*x*_ *exposure and risk of cardiovascular-related malformations*. Squares indicate study-specific risk estimates (size of the square reflects the study-specific statistical weight); horizontal lines indicate 95% CIs; diamond indicates the summary relative risk with its 95% CI.

Nine studies [7, 27–34] were included in the analysis of the association between NO_*x*_ concentrations and cardiovascular-related malformation (Figure 2, Table 1). Overall, the SOR of cardiovascular-related malformation per 10 ppb increment in NO_*x*_ concentration was 1.01 (95% CI: 0.98–1.04), with low heterogeneity (*I*^2^ = 38.6%, *P* = 0.09). There was no indication of a publication bias according to Begg's test (*P-bias* = 1.00) and Egger's test (*P-bias* = 0.65).

**Figure 3:**
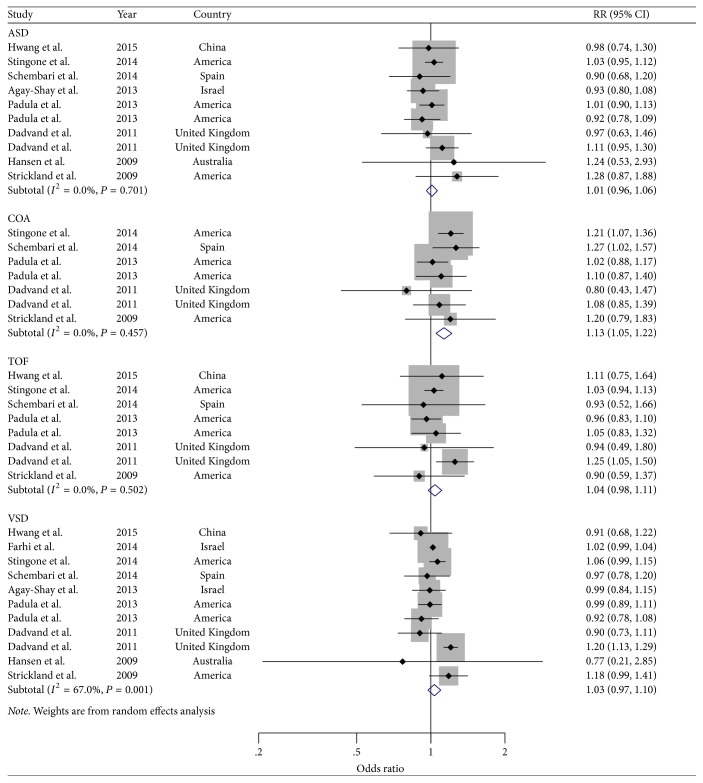
*Forest plots of the relationship between NO*_*x*_ *exposure and risk of ASD, COA, TOF, and VSD*. Squares indicate study-specific risk estimates (size of the square reflects the study-specific statistical weight); horizontal lines indicate 95% CIs; diamond indicates the summary relative risk with its 95% CI.

Furthermore, analyses of studies reported the cardiac malformation of interest that were ASD [7, 27, 28, 30–34], VSD [7, 27–34], TOF [27, 29–32, 34], and COA [29–32, 34] with corresponding OR scores of 1.01 (95% CI: 0.96–1.06), 1.03 (95% CI: 0.97–1.10), 1.04 (95% CI: 0.98–1.11), and 1.13 (95% CI: 1.05–1.22), respectively (Figure 3).

The results of stratified analyses by study characteristics are summarized in Table 1. When we performed subgroup analyses in terms of study design, geographic locations, and confounded adjustments, all strata showed negative results in the outcome of cardiovascular-related malformation and ASD, which were consistent with the main findings. As for the outcome of VSD stratified by study design, the point estimate for cohort study (SOR: 1.02; 95% CI: 1.00–1.05) was slightly higher than that for case-control study (SOR: 1.01; 95% CI: 0.93–1.09), which suggested a mild positive association between NO_*x*_ exposure and VSD in cohort studies. Stratifying by adjustment for educational level, we observed significant results for cardiovascular-related malformation with low heterogeneity after summarizing three studies adjusted for this potential confounder. In addition, there was no evidence of significant heterogeneity between subgroups detected by meta-regression analyses.

In a sensitivity analysis, we evaluated the effect of removing a single study from the total in order to determine its effect on the summarized estimate for heterogeneity and to assess whether one study had a significant influence on the meta-analytic OR. The study-specific OR of cardiovascular-related malformation ranged from a low value of 1.00 (95% CI: 0.90–1.02; *I*^2^ = 0.0%, *P* = 0.56) after omission of the study by Stingone et al. [31] to a high value of 1.01 (95% CI: 0.99–1.04; *I*^2^ = 16.2%, *P* = 0.30) after omission of the study by Padula et al. [30].

### 3.5. NO_2_ and Cardiovascular-Related Malformation per 10 ppb Increment

**Figure 4:**
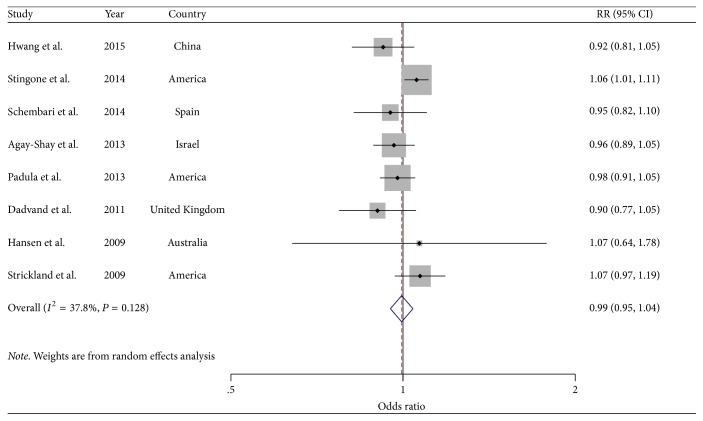
*Forest plots of the relationship between NO*_2_ *exposure and risk of cardiovascular-related malformations*. Squares indicate study-specific risk estimates (size of the square reflects the study-specific statistical weight); horizontal lines indicate 95% CIs; diamond indicates the summary relative risk with its 95% CI.

Eight studies [7, 27–32, 34] were included in the analysis of the association between NO_2_ concentration and cardiovascular-related malformation (Figure 4, Table 2). Overall, the SOR of cardiovascular-related malformation per 10 ppb increment in NO_2_ concentration was 0.99 (95% CI: 0.95–1.04), with low heterogeneity (*I*^2^ = 37.8%, *P* = 0.13). There was no indication of publication bias with Egger's test (*P-bias* = 0.39) and Begg's test (*P-bias* = 0.20).

**Figure 5:**
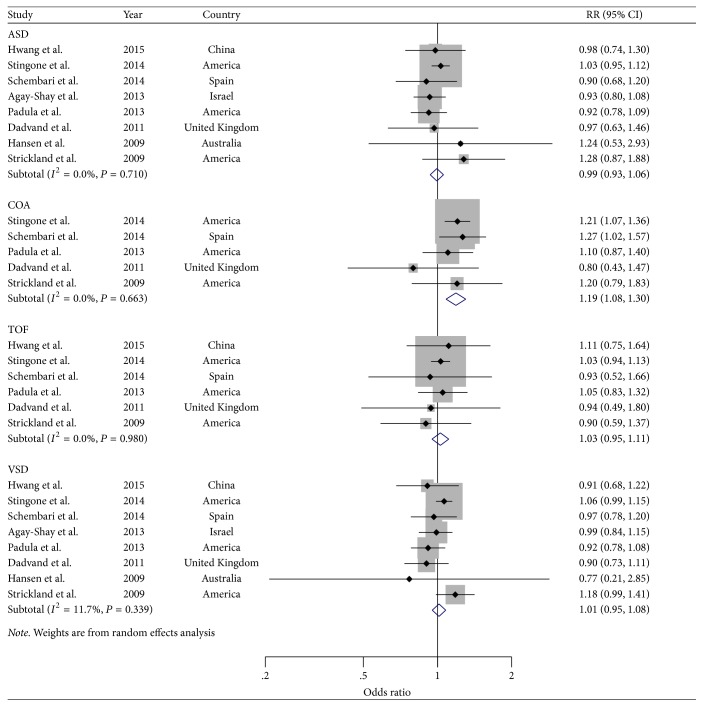
*Forest plots of the relationship between NO*_2_ *exposure and risk of ASD, COA, TOF, and VSD*. Squares indicate study-specific risk estimates (size of the square reflects the study-specific statistical weight); horizontal lines indicate 95% CIs; diamond indicates the summary relative risk with its 95% CI.

In addition, analyses of studies reported the cardiac malformations of interest that were ASD [7, 27–32, 34], VSD [7, 27–32, 34], TOF [27, 29–32, 34], and COA [29–32, 34] with corresponding OR scores of 0.99 (95% CI: 0.93–1.06), 1.01 (95% CI: 0.95–1.08), 1.03 (95% CI: 0.95–1.11), and 1.19 (95% CI: 1.08–1.30), respectively (Figure 5). Of note, there was a significant increase risk of COA in infants born to mothers who are exposed to NO_2_ in the first trimester.

Stratifying by study design, geographic locations, and confounded adjustments, the majority of strata showed negative results in the outcome of cardiovascular-related malformation, ASD, and VSD, which were consistent with the main findings. There was no evidence of significant heterogeneity between subgroups detected by meta-regression analyses. In a sensitivity analysis omitting one study at a time and after we analyzed the SOR of the rest, the SORs of cardiovascular-related malformation ranged from 0.98 (95% CI: 0.94–1.02; *I*^2^ = 0.0%, *P* = 0.51) after excluding the study by Stingone et al. [31] to 1.00 (95% CI: 0.96–1.05; *I*^2^ = 34.6%, *P* = 0.16) after excluding the study by Dadvand et al. [32].

## 4. Discussion

Overall, the findings from this meta-analysis indicated there was no obvious increased risk between NO_*x*_/NO_2_ exposure and cardiovascular-related malformations. The same negative results were also observed in most subgroup analyses. However, we found that exposure to NO_*x*_ and NO_2_ in the first trimester increased the risk for COA malformation by 13% (1.05–1.22) and 19% (95% CI: 1.08–1.30), respectively.

The cause and mechanism of cardiovascular-related malformation still remain unclear. A study conducted by Wilhelm and Ritz [37] suggested that NO_2_ could oxidize tissue composition, increase lipid peroxidation in fetal and maternal vivo, and inhibit the protective action of the antioxidant defense system, which can affect fetal growth and development. Additionally, there was evidence that air pollution could also contribute to epigenetic change, including alteration of DNA methylation [38]. Besides, microRNA has been also studied with regard to the environmental changes and suggested that microRNA expression and regulation may be affected by environmental exposures [39]. These findings pointed out that epigenetic modifications during pregnancy could impair normal embryo development and cause cardiac defects. However, our meta-analysis found no significant increase in risk of most cardiovascular-related malformation with NO_*x*_ or NO_2_ exposure, which might result from exposure assessment of individual study. For example, exposure indices were usually calculated from pollutant measurements at the nearest monitoring station or as a distance-weighted average of measurements of all stations in the area. These methods apply a similar exposure to a relatively large geographic area and thereby measure predominantly community-wide variations in air pollution [18].

Considering traffic exhaust fumes are the main source of NO_*x*_ and NO_2_, this approach is not completely suitable for nitrogen oxide assessment, which may have a much finer spatial distribution. Power may be improved by using more personal measures of NO_*x*_ exposure and the ideal solution is to set monitors in a cohort of pregnant women, but this method would be very expensive [28]. A cheaper alternative is to measure the road network surrounding the pregnancy's home based on their geocoded address, as a proxy measure of pollution [28]. This raises an important issue and may guide further study design and exposure assessment.

It is possible that NO_*x*_ exposure might be associated with cardiovascular-related malformations, but this association is too small to detect because there were several known and unknown factors that might influence the results of individual study. Considering each included study was based on the data from registry or hospital program, the condition of personal exposure might be unpredictable and could be related to specific factors such as behavior pattern, living activity, working history, and indoor air pollution. Nondifferential errors were assumed between cases and controls, and therefore this exposure misclassification could influence effect estimate. For example, if mothers of included cases had more difficult pregnancies, this could limit their outdoor movement [31]. Besides, another plausible explanation of information bias is residential mobility during pregnancy, which may lead to exposure misclassification. The random migration in cases and controls might generate nondifferential misclassification and decrease the accuracy of exposure assessment [27]. These reasons would more likely result in underestimation of NO_*x*_ exposure effects rather than positive results in the association.

Compared to the limited sample size of each study, our meta-analysis included a large number of cardiovascular-related malformations cases (*n* = 19,079). This large sample size not only allowed us to investigate the association between NO_*x*_ exposure and cardiovascular-related malformations, but also facilitated for us carrying out numerous stratified and sensitivity analyses to explore the heterogeneity. Additionally, we only included the studies whose exposure period of pregnancy was first trimester or in the range of first trimester. This could help reduce the selection and detection bias of the final results.

Despite the clear strengths of this study, some limitations of our study should be acknowledged. First of all, we could not get detailed information on diagnostic tests for all cardiovascular-related malformations of included studies. Although echocardiography has been the most useful diagnostic test to confirm the presence of congenital heart defects [40], it greatly depended on the clinical skills and knowledge of operators, which might generate bias in different studies. Besides, all included studies are retrospective. The lack of follow-up could lose sight of potential cases of cardiovascular-related malformations and might lead to underestimating the total cases. Additionally, we found differences in inclusion and exclusion of cases in cardiac defects. Studies differed in their approach to classification of the same anomaly. For example, the study conducted by Strickland et al. [29] reclassified each record using a modified version of the International Pediatric and Congenital Cardiac Code implemented in the Society of Thoracic Surgeons Congenital Heart Surgery Database, while study conducted by Hwang et al. [27] classified the cardiac defect into six categories which were similar with categories used by Gilboa and colleagues [41]. Besides, other studies [7, 28, 30, 32–34] classified the cardiovascular-related malformation according to International Classification of Diseases Tenth Revision (ICD-10). This could become the largest evidence for heterogeneity for cardiac defects. Herein, we encourage further studies that should establish consistent classification standard and definition of cardiovascular-related malformation to reduce bias among studies.

Secondly, confounding factors included in the individual studies are an additional concern. In fact, there are some known or suspected risk factors for congenital anomalies including maternal age, smoking, and season of conception [42–44]. However, these potential confounders were not consistent in each study. For example, Hansen et al. [28] only noted the adjustment for neonate sex while Hwang et al. [27] and Farhi et al. [33] adjusted for seven kinds of potential confounders, respectively. Notably, some specific confounding factors such as maternal body mass index (BMI) and obesity seem to increase the risk of congenital heart defects including the septal ones [45–47]. Therefore, further studies should fully adjust these potential confounders or report analyses stratified by these risk factors to better be able to rule out residual confounders.

Thirdly, the results of some studies [7, 29, 32] were based on the pregnancies reaching at least 20 weeks' gestation. Given this, these studies reported that their gestational windows of interest spanned weeks 3–7 or weeks 3–8 of pregnancy, because embryological evidence indicated the timing of specific stages of cardiac development, beginning with the migration of cells to form the endocardial tubes and culminating with the septation of the ventricles and outflow tracts in weeks 7 and 8 of development [48]. Although this period is in the range of first trimester, it will lead to underestimating harmful effect of NO_*x*_ or NO_2_. For example, atrioventricular septal defect, Ebstein's anomaly, and tricuspid valve dysplasia can all cause intrauterine congestive heart failure, increasing the risk of intrauterine fetal death [29]. If NO_*x*_ or NO_2_ could increase the risk of these malformations, in turn increasing the risk of abortion before week 20, we could not be able to detect these bad effects on cardiovascular-related malformations. Besides, there is experimental research showing that triggering oxidative stress in diabetic mice can result in apoptosis among migrating neural crest cells, which later results in outflow tract defects [49]. This suggests that it is possible that pollutant-induced oxidative stress in earlier weeks of development can trigger similar disruptions in neural crest cells that later affect development of cardiac structures. Further studies are warranted to explore whether windows of susceptibility to environmental insults coincide with the established stages of heart development.

Finally, in order to determine whether the exposure dose of per 10 ppb increment is too small, we recalculated the SORs of cardiovascular-related malformation per 50 ppb increment in NO_*x*_/NO_2_ concentration. Except for the outcome of COA and NO_2_ exposure, there were no significant risks of other outcomes in infants born to mothers who are exposed to NO_*x*_/NO_2_ in the first trimester. Herein, the exposure dose was not the explanation for our negative results.

## 5. Conclusion

Our meta-analysis provided the limited evidence about the relationship between NO_*x*_ or NO_2_ exposure in pregnant women in the first trimester and cardiovascular-related malformations, although there was a small increase risk between exposure to NO_*x*_/NO_2_ and COA malformation. Additional epidemiologic studies are warranted to provide more detailed results, including research into different kinds of cardiovascular-related malformation after better adjustment for more potential confounders.

## Figures and Tables

**Table 1:** Summary risk estimates of the associations between NO*x* and cardiovascular-related malformation, ASD, and VSD.

	Cardiovascular-related malformation					ASD					VSD				
	Number of studies	SOR (95% CI)	*I* ^2^ (%)	*P* ^*∗*^	*P* ^†^	Number of studies	SOR (95% CI)	*I* ^2^ (%)	*P* ^*∗*^	*P* ^†^	Number of studies	SOR (95% CI)	*I* ^2^ (%)	*P* ^*∗*^	*P* ^†^
*Overall*	9	1.01 (0.98–1.04)	38.6	0.09		8	1.01 (0.96–1.06)	0	0.70		9	1.03 (0.97–1.10)	67.0	<0.01	
*Study design*					0.86					0.55					0.59
Cohort study	3	1.01 (0.99–1.03)	0	0.73		2	1.04 (0.77–1.39)	55.6	0.13		3	1.02 (1.00–1.05)	0	0.44	
Case-control study	6	1.03 (1.01–1.05)	0	0.57		6	1.02 (0.96–1.08)	0	0.81		6	1.01 (0.93–1.09)	57	0.01	
*Geographic location*					0.27					0.79					0.88
US	3	1.03 (1.01–1.06)	0	0.73		3	1.02 (0.95–1.08)	0	0.43		3	1.04 (0.98–1.1)	0	0.53	
Others	6	1.01 (0.99–1.03)	0	0.50		5	1.00 (0.91–1.10)	0	0.61		6	1.03 (0.93–1.13)	76	<0.01	
*Adjustment for confounders*															
*Maternal age *					0.51					0.59					0.63
Yes	5	1.02 (0.99–1.06)	30.7	0.10		4	1.00 (0.93–1.07)	0	0.59		5	1.02 (1.00–1.05)	0	0.71	
No	4	1.02 (0.99–1.05)	0	0.92		4	1.03 (0.95–1.11)	0	0.52		4	1.03 (0.93–1.14)	56.8	0.01	
*Socioeconomic status*					0.06					0.78					0.51
Yes	5	1.02 (0.99–1.06)	32.0	0.09		5	1.02 (0.95–1.08)	0	0.61		5	1.04 (0.94–1.15)	69.3	<0.01	
No	4	1.01 (0.99–1.03)	0	0.98		3	1.00 (0.91–1.10)	0	0.44		4	1.02 (1.00–1.04)	0	0.71	
*Conception season*					0.26					0.75					0.72
Yes	5	1.01 (0.98–1.03)	4.1	0.41		4	1.00 (0.91–1.09)	0	0.51		5	1.03 (0.93–1.13)	79.8	<0.01	
No	4	1.03 (1.01–1.06)	0	0.76		4	1.02 (0.96–1.08)	0	0.57		4	1.04 (0.98–1.10)	0	0.61	
*Year of birth*					0.02					0.72					0.33
Yes	4	1.03 (0.99–1.07)	41.3	0.06		4	1.02 (0.95–1.08)	0	0.47		4	1.05 (0.95–1.16)	72.6	0.01	
No	5	1.01 (0.99–1.02)	0	0.98		4	1.00 (0.91–1.09)	0	0.61		5	1.02 (0.99–1.04)	0	0.74	
*Infant sex*					0.44					0.6					0.72
Yes	3	1.01 (0.99–1.03)	0	0.78		2	0.94 (0.81–1.09)	0	0.52		3	1.02 (1.00–1.04)	0	0.86	
No	6	1.03 (1.01–1.05)	0	0.61		6	1.02 (0.97–1.08)	0	0.68		6	1.03 (0.96–1.11)	54	0.01	
*Gestational age*					N/A					N/A					N/A
Yes	1	0.92 (0.81–1.05)	N/A	0.70		1	0.98 (0.74–1.30)	N/A	N/A		1	0.91 (0.68–1.22)	N/A	N/A	
No	8	1.02 (1.01–1.03)	0	0.65		7	1.01 (0.96–1.07)	0	0.61		8	1.04 (0.98–1.10)	57.1	<0.01	
*Educational level*					0.56					0.95					0.29
Yes	3	1.02 (1.00–1.03)	0	0.63		2	1.01 (0.95–1.08)	0	0.51		3	1.02 (1.00–1.04)	0	0.69	
No	6	1.02 (0.98–1.06)	0	0.53		6	1.01 (0.93–1.11)	0	0.54		6	1.05 (0.95–1.17)	55.2	0.03	

^*∗*^ *P* value for heterogeneity within each subgroup; ^†^*P* value for heterogeneity between subgroups with meta-regression analysis. ASD, atrial septal defect; CI: confidence interval; SOR, summary odds ratio; VSD, ventricular septal defect.

**Table 2:** Summary risk estimates of the associations between NO_2_ and cardiovascular-related malformation, ASD, and VSD.

	Cardiovascular-related malformation					ASD					VSD				
	Number of studies	SOR (95% CI)	*I* ^2^ (%)	*P* ^*∗*^	*P* ^†^	Number of studies	SOR (95% CI)	*I* ^2^ (%)	*P* ^*∗*^	*P* ^†^	Number of studies	SOR (95% CI)	*I* ^2^ (%)	*P* ^*∗*^	*P* ^†^
*Overall*	8	0.99 (0.95–1.04)	37.8	0.13		8	0.99 (0.93–1.06)	0	0.71		8	1.01 (0.95–1.08)	11.7	0.34	
*Study design*					0.91					0.80					0.27
Cohort study	2	1.01 (0.94–1.07)	0	0.66		2	1.04 (0.77–1.39)	55.6	0.13		2	1.07 (0.95–1.21)	5.8	0.35	
Case-control study	6	1.02 (0.99–1.05)	10.4	0.29		6	1.00 (0.93–1.07)	0	0.82		6	1.01 (0.95–1.07)	0	0.60	
*Geographic location*					0.01					0.31					0.14
US	3	1.05 (1.02–1.08)	0	0.48		3	1.01 (0.91–1.13)	26.9	0.26		3	1.05 (0.99–1.12)	0	0.43	
Others	5	0.95 (0.89–1.00)	0	0.93		5	0.94 (0.84–1.06)	0	0.96		5	0.95 (0.86–1.05)	0	0.95	
*Adjustment for confounders*															
*Maternal age*					0.61					0.91					0.57
Yes	4	1.02 (0.98–1.06)	28.1	0.13		4	1.00 (0.93–1.07)	0	0.59		4	1.04 (0.97–1.10)	0	0.58	
No	4	1.00 (0.94–1.05)	0	0.79		4	0.98 (0.85–1.13)	0	0.46		4	0.99 (0.89–1.10)	0	0.44	
*Socioeconomic status*					0.96					0.94					0.83
Yes	5	1.01 (0.97–1.05)	32.6	0.09		5	1.00 (0.93–1.07)	0	0.75		5	1.02 (0.96–1.09)	0	0.48	
No	3	1.01 (0.95–1.07)	0	0.82		3	1.02 (0.82–1.28)	23	0.27		3	1.02 (0.91–1.15)	0	0.44	
*Conception season*					0.01					0.27					0.14
Yes	4	0.94 (0.89–1.00)	0	0.96		4	0.94 (0.83–1.05)	0	0.98		4	0.95 (0.86–1.06)	0	0.89	
No	4	1.05 (1.02–1.08)	0	0.54		4	1.02 (0.95–1.10)	0	0.40		4	1.05 (0.99–1.12)	0	0.53	
*Year of birth*					0.44					0.92					0.89
Yes	4	1.02 (0.98–1.07)	43.9	0.05		4	1.00 (0.93–1.07)	0	0.59		4	1.03 (0.97–1.09)	0	0.41	
No	4	0.99 (0.94–1.05)	0	0.85		4	0.98 (0.86–1.12)	0	0.46		4	1.01 (0.90–1.12)	0	0.51	
*Infant sex*					0.35					0.43					0.76
Yes	2	0.97 (0.89–1.05)	0	0.80		2	0.94 (0.81–1.09)	0	0.52		2	0.99 (0.84–1.15)	0	0.71	
No	6	1.03 (1.00–1.06)	6.9	0.34		6	1.01 (0.94–1.08)	0	0.63		6	1.03 (0.97–1.09)	0	0.44	
*Gestational age*					N/A					N/A					N/A
Yes	1	0.92 (0.81–1.05)	N/A	0.70		1	0.98 (0.74–1.30)	N/A	N/A		1	0.91 (0.68–1.22)	N/A	N/A	
No	7	1.03 (1.00–1.06)	2.3	0.43		7	1.00 (0.93–1.06)	0	0.60		7	1.03 (0.97–1.09)	0	0.57	
*Educational level*					0.07					0.54					0.83
Yes	2	1.04 (1.01–1.08)	10.5	0.32		2	1.00 (0.91–1.10)	25.7	0.25		2	1.03 (0.96–1.11)	1.9	0.38	
No	6	0.98 (0.93–1.03)	0	0.79		6	0.97 (0.86–1.08)	0	0.73		6	1.00 (0.92–1.09)	0	0.56	

^*∗*^ *P* value for heterogeneity within each subgroup; ^†^*P* value for heterogeneity between subgroups with meta-regression analysis. ASD, atrial septal defect; CI: confidence interval; SOR, summary odds ratio; VSD, ventricular septal defect.

## Supplementary Materials

Supplementary Materials Supplementary Table S1: characteristics of the studies included in the meta-analysis. Supplementary Table S2: adjusted factors of the studies included in the meta-analysis. Supplementary Table S3a: methodological quality of case-control studies included in the meta-analysis. Supplementary Table S3b: methodological quality of cohort studies included in the meta-analysis.
