# Supplementary material for: Exposure to Nitrogen Oxide in the First Trimester and Risk of Cardiovascular-Related Malformations: A Dose-Response Meta-Analysis of Observational Studies
Source: Biomed Res Int. 2018 Apr 10;2018:1948407. doi: 10.1155/2018/1948407 (PMC5914127; doi:10.1155/2018/1948407)
Supplement: Supplementary Materials — Supplementary Table S1: characteristics of the studies included in the meta-analysis. Supplementary Table S2: adjusted factors of the studies included in the meta-analysis. Supplementary Table S3a: methodological quality of case-control studies included in the meta-analysis. Supplementary Table S3b: methodological quality of cohort studies included in the meta-analysis. [file 1948407.f1.pdf]

**Exposure to nitrogen oxide in the first trimester and risk of cardiovascular-related malformations: a dose-response meta-analysis of observational studies.**

Tie-Ning Zhang[1], Da Li[2], Qi-Jun Wu[3], Jing Xia[1], Ri Wen[1], Xing-Chen Chen[4], Ni Yang[1], Yan-Ling Chen[5], Yan-Hong Huang[6], Chun-Feng Liu[1].

**Supplementary Table S1. Characteristics of the studies included in the meta-analysis.**

| First author (ref), year, location | Study period | Study design | Gestational period | Exposure | Outcomes (No. of cases) | Risk estimates (95% CI) |
|---|---|---|---|---|---|---|
| Hwang (27), 2015, China | 2001-2007 | Case-control study | First trimester | $NO_2$ | ASD (194) | 0.98 (0.74-1.30) |
| | | | | | Conotruncal Defects (404) | 0.81 (0.60-1.10) |
| | | | | | PDA (213) | 1.02 (0.78-1.33) |
| | | | | | Pulmonary Artery and Valve (60) | 0.77 (0.47-1.27) |
| | | | | | TOF (111) | 1.11 (0.75-1.64) |
| | | | | | Transposition of the Great Arteries (70) | 0.72 (0.44-1.16) |
| | | | | | VSD (193) | 0.91 (0.68-1.22) |
| Farhi (33), 2014, Israel | 1997-2004 | Historical cohort-study | First trimester | $NO_x$ | Circulatory system (1,634) | 1.01 (0.99-1.03) |
| | | | | | VSD (1,161) | 1.02 (0.99-1.04) |
| Schembari (34), 2014, Spain* | 1994-2006 | Case-control study | Week 3-8 | $NO_2$ | ASD (229) | 1.20 (1.02-1.41) |
| | | | | | COA (69) | 1.13 (0.90-1.41) |
| | | | | | CHD (823) | 1.36 (1.06-1.75) |
| | | | | | TOF (49) | 1.11 (0.75-1.64) |
| | | | | | VSD (351) | 0.91 (0.68-1.22) |

**Supplementary Table S1. (Continued)**

| First author (ref), year, location | Study period | Study design | Gestational period | Exposure | Outcomes (No. of cases) | Risk estimates (95% CI) |
|---|---|---|---|---|---|---|
| Stingone (31),2014, America | 1997-2006 | Case-control study | Week 2-8 | $NO_2$ | LVOTO (545) | 1.07 (1.00-1.16) |
| | | | | | COA (215) | 1.21 (1.07-1.36) |
| | | | | | Conotruncal (691) | 1.05 (0.98-1.12) |
| | | | | | TOF (355) | 1.03 (0.94-1.13) |
| | | | | | APVR (100) | 0.95 (0.83-1.09) |
| | | | | | AVSD (53) | 0.92 (0.76-1.11) |
| | | | | | RVOTO (459) | 1.16 (1.07-1.25) |
| | | | | | Septal (944) | 1.05 (0.99-1.12) |
| | | | | | ASD (461) | 1.03 (0.95-1.12) |
| | | | | | VSD (395) | 1.06 (0.99-1.15) |
| Agay-Shay (7), 2013, Israel | 2000-2006 | Registry-based cohort study | Week 3-8 | $NO_2$ | Isolated atrial and ASD (534) | 0.93 (0.80-1.08) |
| | | | | | Isolated PDA (147) | 1.03 (0.77-1.38) |
| | | | | | Isolated VSD (493) | 0.99 (0.84-1.15) |
| | | | | | Multiple CHD (569) | 0.96 (0.83-1.11) |

**Supplementary Table S1. (Continued)**

| First author (ref), year, location | Study period | Study design | Gestational period | Exposure | Outcomes (No. of cases) | Risk estimates (95% CI) |
|---|---|---|---|---|---|---|
| Padula (30), 2013, America* | 1997– 2006 | Case-control study | First 2 months of pregnancy | NO | Aortic stenosis (23) | 1.04 (0.81-1.34) |
| | | | | | COA (70) | 1.02 (0.88-1.17) |
| | | | | | Dextro-transposition of the great arteries (54) | 0.94 (0.79-1.11) |
| | | | | | Double outlet right ventricle – other (14) | 0.91 (0.63-1.33) |
| | | | | | Heterotaxia (54) | 0.97 (0.83-1.14) |
| | | | | | Hypoplastic left heart syndrome (53) | 1.03 (0.87-1.21) |
| | | | | | Pulmonary atresia (50) | 0.99 (0.84-1.18) |
| | | | | | Secundum ASD (123) | 1.01 (0.90-1.14) |
| | | | | | TOF (88) | 0.96 (0.83-1.10) |
| | | | | | Total anomalous pulmonary venous return (19) | 0.95 (0.72-1.25) |
| | | | | | VSD-muscular (46) | 0.95 (0.79-1.14) |
| | | | | | VSD-perimembranous (88) | 1.02 (0.89-1.17) |

**Supplementary Table S1. (Continued)**

| First author (ref), year, location | Study period | Study design | Gestational period | Exposure | Outcomes (No. of cases) | Risk estimates (95% CI) |
|---|---|---|---|---|---|---|
| | | | | $NO_2$ | Aortic stenosis (26) | 0.86 (0.59-1.25) |
| | | | | | Atrioventricular septal defects (14) | 1.00 (0.59-1.71) |
| | | | | | COA (78) | 1.10 (0.87-1.40) |
| | | | | | Dextro-transposition of the great arteries (63) | 1.25 (0.96-1.62) |
| | | | | | Double outlet right ventricle – TGA (17) | 1.19 (0.79-1.81) |
| | | | | | Heterotaxia (61) | 0.99 (0.77-1.26) |
| | | | | | Hypoplastic left heart syndrome (61) | 0.90 (0.69-1.18) |
| | | | | | Pulmonary atresia (59) | 0.95 (0.73-1.24) |
| | | | | | Secundum ASD (140) | 0.92 (0.78-1.09) |
| | | | | | Single ventricle or other complex (21) | 0.86 (0.59-1.25) |
| | | | | | TOF (100) | 1.05 (0.83-1.32) |
| | | | | | Tricuspid atresia (18) | 0.88 (0.53-1.46) |
| | | | | | VSD-conov (19) | 1.02 (0.68-1.54) |
| | | | | | VSD-muscular (48) | 0.90 (0.69-1.16) |
| | | | | | VSD-perimembranous (98) | 0.90 (0.71-1.14) |

**Supplementary Table S1. (Continued)**

| First author (ref), year, location | Study period | Study design | Gestational period | Exposure | Outcomes (No. of cases) | Risk estimates (95% CI) |
|---|---|---|---|---|---|---|
| Dadvand (32), 2011, United Kingdom* | 1993–2003 | Case-control study | Week 3-8 | NO | Pooled-cases (2,140) | 1.07 (1.01-1.13) |
| | | | | | ASD (274) | 1.11 (0.95-1.30) |
| | | | | | COA (125) | 1.08 (0.85-1.39) |
| | | | | | TOF (126) | 1.25 (1.05-1.50) |
| | | | | | VSD (1,154) | 1.20 (1.13-1.29) |
| | | | | $NO_2$ | Pooled-cases (2,140) | 0.90 (0.77-1.05) |
| | | | | | ASD (274) | 0.97 (0.63-1.46) |
| | | | | | COA (125) | 0.80 (0.43-1.47) |
| | | | | | TOF (126) | 0.94 (0.49-1.80) |
| | | | | | VSD (1,154) | 0.90 (0.73-1.11) |
| Hansen (28), 2009, Australia* | 1998-2004 | Case-control study | Week 3-8 | $NO_2$ | Aortic artery and valve defect (63) | 1.58 (0.40-6.15) |
| | | | | | ASD (127) | 1.24 (0.53-2.93) |
| | | | | | Conotruncal Defects (63) | 0.30 (0.07-1.34) |
| | | | | | Endocardial cushion and mitral valve defect (33) | 3.04 (0.51-18.06) |
| | | | | | Pulmonary artery and valve defect (64) | 1.10 (0.29-4.15) |
| | | | | | VSD (222) | 0.77 (0.21-2.85) |

**Supplementary Table S1. (Continued)**

| First author (ref), year, location | Study period | Study design | Gestational period | Exposure | Outcomes (No. of cases) | Risk estimates (95% CI) |
|---|---|---|---|---|---|---|
| Strickland (29), 2009, America* | 1986-2003 | Retrospective cohort study | Week 3-7 | $NO_2$ | ASD, secundum (379) | 1.28 (0.87-1.88) |
| | | | | | COA (275) | 1.21 (0.79-1.83) |
| | | | | | Conotruncal defect (661) | 0.91 (0.69-1.21) |
| | | | | | Hypoplastic left heart syndrome (175) | 0.85 (0.49-1.47) |
| | | | | | Left ventricular outflow tract defect (558) | 1.02 (0.75-1.38) |
| | | | | | Patent ductus arteriosus (219) | 1.52 (0.92-2.51) |
| | | | | | Pulmonary stenosis, valvar (312) | 1.02 (0.67-1.54) |
| | | | | | Right ventricular outflow tract defect (421) | 1.04 (0.73-1.47) |
| | | | | | TOF (299) | 0.90 (0.59-1.37) |
| | | | | | Transposition of the great arteries (165) | 0.68 (0.38-1.21) |
| | | | | | VSD, muscular (1,108) | 1.16 (0.93-1.46) |
| | | | | | VSD, perimembranous (546) | 1.21 (0.90-1.65) |

* For dose-response, original data of study was converted into “10 ppb increment” as displayed.

Abbreviations: APVR, anomalous pulmonary venous return; ASD, atrial septal defect; AVSD, atrioventricular septal defect; CHD, congenital heart defect; CI: confidence interval; COA, coarctation of the aorta; LVOTO, left ventricular outflow tract obstructions; RVOTO, right ventricular outflow tract obstructions; TOF, tetralogy of Follot; PDA, patent ductus arteriosus; VSD, ventricular septal defect.

**Supplementary Table S2. Adjusted factors of the studies included in the meta-analysis.**

| First author (ref), year, location | Adjusted factors |
|---|---|
| Hwang (27), 2015, China | Maternal age, diabetes mellitus, plurality, gestational age, SES, and season of conception. |
| Farhi (33), 2014, Israel | Maternal age, maternal ethnicity, maternal country of birth, maternal education, plurality, season of birth and infant gender. |
| Schembari (34), 2014, Spain | Maternal age, conception season, year of birth/termination, socioeconomic index. |
| Stingone (31),2014, America | Maternal race, age, educational attainment, household income, maternal smoking status, and alcohol consumption during early pregnancy, nativity, and site-specific heart defect ratio. |
| Agay-Shay (7), 2013, Israel | Multi-births, maternal age, maternal marital status,maternal origin of birth, infant's sex,religion, year of birth, season of conception, ward-base SES. |
| Padula (30), 2013, America | Maternal race/ethnicity, education and vitamin use (for the month prior to and/or fisrt 2 months of pregnancy). |
| Dadvand (32), 2011, United Kingdom | Year of birth, SES, degree of urbanity, and season of conception. |
| Hansen (28), 2009, Australia | Neonate sex. |

**Supplementary Table S2. Adjusted factors of the studies included in the meta-analysis. (Continued)**

| First author (ref), year, location | Adjusted factors |
|---|---|
| Strickland (29), 2009, America | Week of year, and cubic spline for day of follow-up. |

Abbreviations: SES, socioeconomic status.

**Supplementary Table S3. Methodological quality of case-control studies included in the meta-analysis***

| First author (reference), publication year | Case definition adequate | Representativeness of the cases | Selection of Controls | Definition of Controls | Comparability of cases and controls on the basis of the design or analysis† | Ascertainment of exposure | Same method of ascertainment for cases and controls | Non-Response rate‡ |
|---|---|---|---|---|---|---|---|---|
| Hwang (27), 2015 | * | * | * | * | ** | * | * | * |
| Schembari (34), 2014 | * | * | * | * | ** | * | * | - |
| Stingone (31), 2014 | * | * | * | * | ** | * | * | * |
| Padula (30), 2013 | * | * | * | * | - | * | * | * |
| Dadvand (32), 2011 | * | * | * | * | * | * | * | - |
| Hansen (28), 2009 | * | * | * | * | - | * | * | - |

* A study could be awarded a maximum of one star for each item except for the item Control for important factor or additional factor. The definition/explanation of each column of the Newcastle-Ottawa Scale is available from (http://www.ohri.ca/programs/clinical_epidemiology/oxford.asp.).

† A maximum of 2 stars could be awarded for this item. Studies that controlled for maternal age, whereas studies that controlled for socioeconomic status received an additional star.

‡ A case-control study with a non-response rate <10% was assigned one star.

**Supplementary Table S3. Methodological quality of cohort studies included in the meta-analysis** [*]

| First author (reference), publication year | Representativeness of the exposed cohort | Selection of the unexposed cohort | Ascertainment of exposure | Outcome of interest not present at start of study | Control for important factor or additional factor [†] | Assessment of outcome | Follow-up long enough for outcomes to occur [‡] | Adequacy of follow-up of cohorts[§] |
|---|---|---|---|---|---|---|---|---|
| Farhi (33), 2014 | * | * | * | * | * | * | - | - |
| Agay-Shay (7), 2013 | * | * | * | * | ** | * | - | - |
| Strickland (29), 2009 | * | * | * | * | - | * | - | - |

[*] A study could be awarded a maximum of one star for each item except for the item Control for important factor or additional factor. The definition/explanation of each column of the Newcastle-Ottawa Scale is available from (http://www.ohri.ca/programs/clinical_epidemiology/oxford.asp.).

[†] A maximum of 2 stars could be awarded for this item. Studies that controlled for maternal age, whereas studies that controlled for socioeconomic status received an additional star.

[‡] A cohort study with a follow-up time >1year was assigned one star.

[§] A cohort study with a follow-up rate >80% was assigned one star.
